# Comparison of Transcutaneous Electric Nerve Stimulation (TENS) and Microcurrent Nerve Stimulation (MENS) in the Management of Masticatory Muscle Pain: A Comparative Study

**DOI:** 10.1155/2019/8291624

**Published:** 2019-11-23

**Authors:** B. Saranya, Junaid Ahmed, Nandita Shenoy, Ravikiran Ongole, Nanditha Sujir, Srikant Natarajan

**Affiliations:** ^1^Department of Oral Medicine and Radiology, Manipal College of Dental Sciences, Mangalore 575001, India; ^2^Manipal Academy of Higher Education, Manipal 576104, Karnataka, India; ^3^Department of Oral Pathology, Manipal College of Dental Sciences, Mangalore 575001, India

## Abstract

**Introduction:**

Temporomandibular disorders (TMDs) are a heterogeneous group of pathologies affecting the temporomandibular joint (TMJ), the jaw muscles, or both. Epidemiological studies of TMD reveal a prevalence of 82% in the general population with 48% of them presenting with clinical features of muscle tenderness and difficulty in mouth opening. TMD are considered to be the most common orofacial pain conditions of nondental origin.

**Methods:**

The patients with TMD were randomly divided into two groups, A and B, based on their VAS scale. Group A consists of two subgroups 1 and 2 each consisting of 15 patients. Group B consists of two subgroups 3 and 4 consisting of 15 patients. Patients in Group A were given TENS for twenty minutes, and the frequency is adjusted as follows: (i) subgroup 1: TENS frequency at a range of 0–5 (VAS measuring 1–5) and (ii) subgroup 2: TENS frequency at a range of 5 and above (VAS measuring 6–10). Patients in Group B were given MENS for twenty minutes, and the frequency adjusted as follows: (i) subgroup 3: MENS frequency at range of 0–5 (VAS measuring 1–5) and (ii) subgroup 4: MENS frequency at a range of 5 and above (VAS measuring 6–10). Each patient was recalled for five consecutive days for the treatment, and the same intensity and frequency were maintained throughout the treatment period.

**Results:**

The improvement in VAS is seen to be highly significant statistically in MENS subgroup 4 (moderate-to-severe pain). Subgroups 1 and 3 had improvement in VAS which was comparable in both TENS and MENS groups.

**Conclusion:**

In the present study, it was found that TENS and MENS are equally effective in improving the functional mouth opening. MENS showed better and immediate effect in relief of pain. Microcurrent also has the advantage of being subthreshold, and hence the side effects such as tingling sensation and paresthesia seen to occur in some patients following TENS are absent. TENS and MENS can be considered as the first line of treatment in patients with acute and chronic masticatory muscle pain and also as an effective treatment option in cases of functional mouth opening.

## 1. Introduction

Temporomandibular disorders (TMDs) are a heterogeneous group of pathologies affecting the temporomandibular joint (TMJ), the jaw muscles, or both. Epidemiological studies of TMD reveal a prevalence of 82% in the general population, and 48% of them presented with clinical features of muscle tenderness and difficulty in mouth opening. TMDs are considered to be the most common orofacial pain conditions of nondental origin. The frequent concurrent presence of other symptoms such as earache, headache, neuralgia, and tooth pain which may be related to TMD or present as ancillary findings makes the assessment of TMD a complex issue [1].

Myofascial pain syndrome (MPS) is diagnosed in nearly a third of patients who have musculoskeletal pain disorders. Accurate diagnosis allows for appropriate therapy whether it is nonsurgical or surgical. Current trends favor conservative (nonsurgical) therapy, and the surgical interventions have become less aggressive, moving away from open arthroplasty towards arthroscopic procedures [2]. The interrelationship and association of TMDs with various disorders continue to be explored [3]. A number of successful conservative treatment options have been tried for myofascial pain including occlusal splints, physiotherapy, muscle relaxing appliances, pharmacological interventions, physical agents such as thermography, cryotherapy, and ultrasound, complementary and alternative medicine such as acupuncture, and electrotherapy modalities such as transcutaneous electric nerve stimulation (TENS) and microcurrent electric nerve stimulation (MENS) [4]. MENS is a relatively new approach for pain relief and muscle healing, while TENS has been used for pain relief since the sixteenth century.

The use of TENS is based on several interrelated theories on the mechanism of pain transmission and the blocking of those mechanisms. The first one being the gate control theory, the second theory is related to endogenous release of morphine-like substances (endorphin) after electrical stimulation. The third mechanism of action of TENS is related to the automatic and involuntary contraction of muscles.

MENS is a form of electrotherapy current that provides subthreshold or subminimal stimulation lower than 1000 microamps (*μ*A). MENS works on the principle of Arndt–Schulz law. It is theorized that healthy tissue is the result of direct flow of electric current throughout our body. Electrical balance is disrupted when the body is injured at a particular site, causing the electric current to change course. The use of microcurrent over the injured site is thought to realign this flow, thus aiding in tissue repair.

The purpose of this study was to compare the effectiveness of transcutaneous electric nerve stimulation (TENS) and microcurrent electrical nerve stimulation (MENS) on patients suffering from myofascial pain.

## 2. Materials and Methods

After obtaining clearance from the Institutional Ethical Committee, the present study was conducted in the Department of Oral Medicine and Radiology, Manipal College of Dental Sciences, Mangalore. A total of 60 patients above the age of 18 years with clinically diagnosed masticatory muscle pain were included in the study if they fulfilled the following criteria:

1. Clinical diagnosis of myofascial pain [4]
2. Muscle tenderness of any of the muscles of mastication
3. A complain of pain with a duration of more than 3 weeks
4. TMJ stiffness and pain
5. Patients of either gender
6. Patients who have given informed consent for the study

The exclusion criteria were as follows:

1. Patients on analgesics or anti-inflammatory medication/physiotherapy/complementary and alternative medicine (CAM) for the same problem
2. Patients with cardiac pacemakers and implanted defibrillators
3. Areas over cancerous lesions
4. Presence of acute infection in the region
5. Patients who are unwilling to be part of the study

Equipments used for treatment:

1. TENS apparatus adjusted to 50 Hz, with a pulse width of 0.5 msec at 0–60 mA
2. MENS apparatus adjusted to 0.5 Hz, 1000 *μ*A

After obtaining subject demographics, a thorough history of the patient was taken. Routine dental checkup of the patient was done and the findings recorded in the proforma. After recording a thorough case history, patients were assessed for TMDs, and clinical diagnosis of muscle pain was established by following the DC/TMD criteria. Radiographic investigations were carried out in cases that raised suspicion of any underlying bony changes of the TMJ. The patients were explained in detail about the treatment protocol and informed consent was obtained. Mouth opening was recorded on the first day before beginning the treatment. The intensity of pain in the affected side was measured by the Visual Analog Scale (VAS) before the beginning of the treatment. The patients were then randomly divided into two groups, Group A and Group B, based on their VAS score.

Group A consists of two subgroups 1 and 2, each consisting of 15 patients. Group B consists of two subgroups 3 and 4 consisting of 15 patients each. The patients were seated in the dental chair, and the electrodes were applied directly on the skin, using a special conducting gel, over the trigger points or in the general area of pain if specific trigger points could not be elicited. Patients in Group A were given TENS for twenty minutes, and the frequency is adjusted as follows:

- Subgroup 1: TENS frequency at VAS range of 0–5
- Subgroup 2: TENS frequency at VAS range above 5

Patients in Group B were given MENS for twenty minutes, and the frequency is adjusted as follows:

- Subgroup 3: MENS frequency at VAS range of 0–5
- Subgroup 4: MENS frequency at VAS range above 5

Each patient was recalled for five consecutive days for the treatment, and the same intensity and frequency were maintained throughout the treatment period.

Patients were given instructions such as supporting their jaw while yawning and while opening their mouth wide. Bilateral chewing pattern and hot fomentation of the affected side were taught as part of jaw exercises. The VAS was measured every day before starting the treatment. The oral rehabilitation such as correction of high points, restoration of decayed tooth, replacement of missing teeth, and extraction of third molars was done if required after the completion of the 5-day treatment. Patients were instructed not to undergo any further treatment for the masticatory muscle pain in the following one month period and were asked to contact the investigator in case of any discomfort or functional limitation. After one month, these patients were recalled, and their VAS and mouth opening were measured. If any of the patients had discomfort or functional limitation, alternate treatments were considered after the 1-month follow-up.

### 2.1. Statistical Method for Analysis

The data were expressed as mean and standard deviation using 2-way ANOVA. The groups were compared using Student's *t*-test, and the intergroup statistics were done using post hoc analysis. Statistical analysis was performed using SPSS 17.0 software.

Level of significance: *α*=0.05.

We compared the *P* value with the level of significance. If *P* < 0.05, we reject the null hypothesis and accept the alternate hypothesis. If *P* > 0.05, we accept the null hypothesis.

## 3. Results

Genderwise distribution and commonly affected side correlation in the study population are described in Tables 1 and 2, respectively.

TENS group reveals an immediate and steady increase in mouth opening during the one-month follow-up period, whereas MENS group reveals a statistically significant increase in mouth opening from day three onwards and during the one month recall, after treatment. The average baseline mouth opening of both the groups was comparable as explained in Table 3.

Comparison of pain response (VAS) between the TENS and MENS group from day zero to day five and one month recall as depicted in Table 4 reveals a decrease in pain more markedly in the MENS group especially after day four of the treatment. Patients in the MENS group also showed a significant immediate positive response to treatment in comparison with the TENS group. However the change in VAS is seen to be comparable in both TENS and MENS group at one month recall.

The inter subgroup comparison in the improvement of mouth opening reveals that the improvement in mouth opening was statistically significant in both TENS and MENS group. However, MENS improves functional mouth opening significantly in patients with moderate-to-severe pain (Group B, subgroup 4) when compared with TENS.

The improvement in pain scale was more marked in subgroup 2 (moderate-to-severe pain) in patients under TENS Therapy. Patients in subgroups 1 and 2 showed a 60% reduction in pain by day 4 of treatment. The pain relief at one month recall in both the subgroups is comparable.

The improvement in VAS was seen to be significant statistically in MENS moderate-to-severe pain group (Group B, subgroup 4). In subgroup 3 of Group B (MENS therapy), the improvement in VAS is comparable with the results achieved in subgroup 1 in patients undergoing TENS therapy (Group A).

## 4. Discussion

TMDs are characterized by a classically described triad of clinical signs: muscle and/or TMJ pain; TMJ sounds; and restriction, deviation, or deflection of the mouth-opening path [5].

There is evidence that the prevalence of TMD signs and symptoms may be high in the general population [6]. The literature reports great variability in the prevalence of the clinical symptoms (6–93%) and signs (0–93%), probably as a result of the different clinical criteria employed. Between 3–7% of the population seek treatment for pain and dysfunction of the TMJ or related structures [7].

In the sample population recruited for our study, the number of female subjects was more than the number of male subjects. There is a statistically significant prevalence of TMDS among females in our patient population. Cairns in 2010 proposed that psychosocial stressors contribute to the development of TMD-related pain, particularly masticatory muscle pain, and hence more women suffer from TMD than men. Although there are arguably multiple reasons for sex-related differences in the prevalence of TMD, one potential trouble shooter for the increased occurrence of this disorder in women has been suggested to be the female sex hormone oestrogen [8].

Among the 60 patients, 50% (30 patients) were affected with pain on the left side, and this finding was statistically significant. We could not attribute this to any of the factors like missing teeth, wear facets, or prosthesis. This was contradictory to the findings of a study undertaken by Diemberger who observed that the right side was the commonly affected side. Their study revealed that women reported more frequently of a preferred chewing side (PCS), and in 64% of the recorded cases, it was observed that the right side (64%) was the preferred chewing side. PCS was found in almost half of the study population and was associated with unilateral signs of TMD, TMJ pain, and asymmetrical loss of antagonist contact [9]. One probable cause for our contradictory finding could be the prevalence of PCS in the left side.

MENS had a statistically significant increase in mouth opening as compared with TENS at the end of the fifth day of the treatment regimen and also at one month recall. However, TENS showed a faster increase in mouth opening compared with MENS. To our knowledge, there are no documented studies comparing improvement in mouth opening between TENS and MENS.

TENS group revealed an acceptable improvement in pain scale by day two, and patients obtained a 43% improvement in pain score by day three of treatment. There was an improvement of pain by 91.02% from the day of initiation of treatment to one month recall. MENS group showed acceptable pain relief from day one itself, and a 60% reduction in pain was obtained in patients by day three. The decrease in pain was seen to be more marked in the MENS group especially after day four of the treatment. Patients in the MENS group showed a significant immediate response to treatment in comparison with the TENS group.

A study by Rajpurohit et al. showed a significant improvement in VAS in the MENS group than patients in the TENS group, in patients with masticatory muscle pain secondary to bruxism, which was in accordance with the findings of our study [10]. Their study, however, does not measure the functional mouth opening. Our study is also the first to consider patients with two different degrees of pain in VAS,mild to moderate and moderate to severe.

## 5. Conclusion and Summary

Physical therapies have been used as an adjunct in the management of chronic and acute masticatory muscle pain of various etiologies. They have various advantages: noninvasive, negligible side effects, not technique sensitive, and easy to use. They form an alternative modality to medicinal management of masticatory muscle pain. However, the efficacy of one physical therapy with another has not been compared in a randomized controlled trial. The present study aims to compare the effectiveness of two physical therapy modalities, namely, TENS & MENS.

In the present study, it was found that TENS and MENS are equally effective in improving the functional mouth opening. However, MENS showed better and immediate effect in relief of pain. Microcurrent also has the advantage of being subthreshold, and hence the side effects such as tingling sensation and paresthesia which are seen to occur in a few patients following TENS are absent in MENS therapy. TENS and MENS can be considered as the first line of treatment in patients with acute and chronic masticatory muscle pain and also as an effective treatment option in cases of functional mouth opening.

The following aspects need to be considered in any future research:

1. TMDs are usually chronic and are seen to recur following periods of remissions, and hence long term follow-ups should be considered.
2. It is important to consider the psychological tangent to TMDs, and hence future studies could include a questionnaire on the patient's anxiety and stress scale, pre- and posttreatment effect on quality of life.

## Figures and Tables

**Table 1:** Genderwise distribution of masticatory muscle pain among the study population. Males are represented by 1 and females by 2.

		Type	
		MENS	TENS	Total
Sex	1	9 30.0%	9 30.0%	18 30.0%
	2	21 70.0%	21 70.0%	42 70.0%
Total		30 100.0%	30 100.0%	60 100.0%

**Table 2:** Affected side distribution among the study population. Right side is represented by 1, left side by 2, and 3 represents both right and left.

		Type	
		MENS	TENS	Total
Sides	1	14 46.7%	9 30.0%	23 38.3%
	2	13 43.3%	17 56.7%	30 50.0%
	3	3 10.0%	4 13.3%	7 11.7%
Total		30 100.0%	30 100.0%	60 100.0%

**Table 3:** Improvement in mouth opening in the study period between the TENS and MENS group (post hoc analysis by Bonferroni test; measure: MEASURE_1; parameter: MOUTH OPENING (MM)).

Type	Mean difference	Std. error	Change (%)	*P* value	
MENS: day 0 to day1	−0.133	0.142	0.45	1.000	
Day 2	−1.233	0.278	4.19	0.003	HS
Day 3	−3.400	0.409	11.55	0.000	HS
Day 4	−5.267	0.452	17.89	0.000	HS
Day 5	−6.800	0.535	23.10	0.000	HS
At 1 month	−7.167	0.601	24.35	0.000	HS

TENS: day 0 to day1	−0.600	0.195	2.02	0.097	
Day2	−1.733	0.442	5.83	0.010	Sig
Day3	−3.200	0.602	10.76	0.000	HS
Day4	−4.000	0.625	13.45	0.000	HS
Day5	−5.000	0.690	16.82	0.000	HS
At 1 month	−5.333	0.914	17.94	0.000	HS

**Table 4:** Improvement in VAS in the study period between the TENS and MENS group (post hoc analysis by Bonferroni test; measure: MEASURE_1; parameter: VAS).

Type	Mean difference	Std. error	Change (%)	*P* value	
MENS: day 0 to day1	0.600	0.163	10.78	0.020	Sig
Day2	1.933	0.197	34.73	0.000	HS
Day3	3.400	0.252	61.08	0.000	HS
Day4	4.733	0.262	85.03	0.000	HS
Day5	5.067	0.262	91.02	0.000	HS
At 1 month	5.067	0.318	91.02	0.000	HS

TENS: day 0 to day1	0.200	0.074	3.68	0.245	
Day2	1.333	0.154	24.54	0.000	HS
Day3	2.333	0.188	42.94	0.000	HS
Day4	3.733	0.271	68.71	0.000	HS
Day5	4.533	0.295	83.44	0.000	HS
At 1 month	4.700	0.319	86.50	0.000	HS

## Data Availability

The data used to support the findings of this article are included within the article.
